# Sociodemographic factors associated with reported attempts at weight loss and specific dietary regimens in Sweden: The SWEDIET-2017 study

**DOI:** 10.1371/journal.pone.0197099

**Published:** 2018-05-10

**Authors:** Linnea Bärebring, Anna Winkvist, Hanna Augustin

**Affiliations:** Department of Internal Medicine and Clinical Nutrition, Sahlgrenska Academy, University of Gothenburg, Gothenburg, Sweden; Dasman Diabetes Institute, KUWAIT

## Abstract

The aim of this study was to investigate the prevalence of active weight loss attempts in Sweden, and to study the extent to which overweight individuals may or may not correctly identify themselves as overweight. Additional aims were to determine the sociodemographic factors associated with following a specific dietary regimen and with attempts at losing weight. A postal questionnaire was sent to 2000 randomly selected men and women living in Sweden. The inclusion criteria was an age of 20–65 years. In total, the response rate was 28% and the completed questionnaires from 555 participants were analyzed in this study. In total, 46% of participants were overweight or obese by self-reported height and weight. Additionally, 42% of overweight and 90% of obese individuals correctly identified themselves as being overweight. Weight loss was pursued by 41% and was more common among women, those with higher physical activity, higher BMI and higher socioeconomic position. Overall, 22% followed a specific diet, and following a dietary regimen was associated with female gender, higher education level and overweight. In conclusion, almost half of the participants were either overweight or trying to lose weight. Trying to lose weight and following a specific dietary regimen were related to female gender, high BMI and higher socioeconomic position. This could indicate that the socioeconomic disparities in health are further exacerbated, as overweight individuals with poor socioeconomic position might be more likely to remain overweight.

## Introduction

The worldwide prevalence of overweight and obesity has increased dramatically during the past decades [1–3]. In 2016, half the adult Swedish population was either overweight or obese by self-reported data [1]. There are socioeconomic gradients to overall health, and Swedish data show that those with lower education have poorer health and higher mortality [1]. It is established that low socioeconomic status is associated with overweight and the prevalence of overweight between 1990–2010 increased more among those with a lower education level in Europe [4]. However, less is known about sociodemographic gradients associated with dieting and attempts at weight loss.

Research shows that weight management practices are often related to dissatisfaction with one’s body [5, 6]. Body size dissatisfaction is in turn associated with overweight and obesity, age, female gender and lower levels of physical activity [7–9]. Findings consistently show that women are less likely to be satisfied with their weight and more likely to have a desire to lose weight, than men [10, 11]. Among those women and men who wish to lose weight, women are less satisfied with their bodies [12]. Though previous studies have demonstrated that a desire to lose weight is more common among those with overweight, many normal weight individuals also wish to lose weight [13]. The term weight misconception is sometimes used to describe a disparity between one’s perceived and actual weight. Weight misconception among overweight individuals is associated with a lower likelihood of attempts at losing weight [14, 15]. However, data on the predictors of attempts at actual weight loss are scarce, especially in Sweden.

A 2010–2011 Swedish national dietary survey showed that women had a lower body mass index (BMI) and less overweight than men [16]. In this survey, 6% of the respondents reported following a specific diet and another 3% reported following a vegetarian or vegan dietary practice [16]. However, the specific diets that were followed were not identified and there are subsequently very few data on how common different dietary regimens are in Sweden. Low carbohydrate diets have been popular in Sweden since the early 2000’s [17], but the prevalence of this type of dietary regimen is unknown.

In summary, there is a scarcity of data from Sweden on the prevalence of specific dietary regimens and how common attempts at weight loss are. Worldwide, there is also very little knowledge on the sociodemographic factors related to weight perception, weight loss and dietary regimens. To our knowledge, no one has previously studied the association between dieting and attempts to lose weight with several sociodemographic factors simultaneously, thus determining the individual impact of these factors. This is especially important as confounding factors are likely to be highly prevalent in analyses relating to weight status. The aim of this study was to identify the proportion of the Swedish population who actively tried to lose weight and to study how many that could correctly identify themselves as overweight. In addition, the aim was to identify sociodemographic factors associated with a desire for weight loss and following a specific dietary regimen.

## Materials and methods

This study was a part of a cross-sectional study of beliefs and attitudes toward diet and health in Sweden—the SWEDIET-2017 survey.

### Recruitment

In January and February 2017, a questionnaire was sent via postal mail to 2000 individuals in Sweden. The individuals were randomly selected from the SPAR registry, which includes addresses of all persons registered as residing in Sweden [18], except those individuals with classified personal information or who lack a registered Swedish address. Individuals aged 20–65 years from all parts of Sweden were invited to take part. Individuals agreeing to participate were instructed to fill in the questionnaire and return it in a pre-addressed envelope. Deadline for submission was July 31^st^ 2017 and questionnaires returned after this date were excluded. No reminders were issued. Questionnaires with over 20% missing data were excluded in the analyses. The study was approved by the Regional Ethics Committee in Gothenburg, Sweden. All participants were informed that returning a filled in questionnaire was regarded as informed consent to participate in the study.

### Data collection

The questionnaire was six pages long, with questions on demographic characteristics, health, dietary choices and restrictions and desire to lose weight. Demographic characteristics included gender, age, income, education and employment. Health data included perceived overweight (Do you have or consider yourself to have overweight: yes or no), self-reported weight (kg) and height (cm). Physical activity was estimated by asking how often the participants engaged in at least 10 minutes of physical exercise that made them sweat (<1/month, 1-3/month, 1/week or ≥1/week). A question was also posed regarding the participants’ desire to lose or gain weight (Are you actively trying to change your weight: no, yes I want to lose weight or yes I want to gain weight). The participants were not provided with a definition of the term “actively trying”.

Participants were asked about their gender (three categories; male, female or other), age (6 categories: 20–24, 25–30, 31–36, 37–45, 46–55, 56–65 years), education (6 levels: primary, secondary 2 years, secondary 3 years, university <3 years, university ≥3 years or other/folk high school/advanced vocational education), monthly income (7 categories in thousands SEK: <10, 10–15, 15–20, 20–25, 25–30, 30–40, ≥40) and employment (6 categories: full time, part time, unemployed, student, on parental leave or other/pensioner).

The participants were further asked if they followed a specific dietary regimen (no, yes). If yes, participants were asked to specify the diet. Eight diets were pre-specified in the survey: low carbohydrate diet, low glycemic index diet, intermittent fasting, raw food, paleolithic diet, weight watchers or similar, vegetarian diet and vegan diet. In addition, respondents could specify any other diet that they followed. This generated the semi-vegetarian diet category that included diets with very low meat consumption (flexitarian) and pesco-vegetarian diets. In addition, the participants were asked how effective they believed the pre-specified diets were for weight loss (five alternatives; very effective, partly effective, partly ineffective, very ineffective or no opinion) and how beneficial the diets were for health (five alternatives; very healthy, partly healthy, partly unhealthy, very unhealthy or no opinion).

### Statistical analyses

Power calculations showed that a sample size of 385 was required to reflect the Swedish population (90% confidence level, 5% margin of error). A total of 2000 individuals were invited to participate, to account for the expected participation rate of 20%.

Being overweight or obese by BMI, calculated using self-reported weight and height, was compared to whether the participants identified as having overweight (answering “yes” to if they considered themselves to have overweight). Those with a calculated BMI ≥25 kg/m^2^ and who also identified as overweight were considered as having correctly identified themselves as overweight.

Determinants of binary outcomes (desire to lose weight and following a specific dietary regimen) were evaluated using multivariable logistic regression analysis. Independent variables included in the models were gender, age, education, monthly income, employment, physical activity and BMI by self-reported height and weight, categorized as <25, 25–30, ≥30 kg/m^2^. Determinants of the desire to gain weight were not studied due to few cases. Chi square test was used to test for differences between categorical variables (proportion of overweight depending on gender). Computer software IBM SPSS Statistics version 22.0 for Windows, (Armonk, NY: IBM Corp.) was used for all statistical analyses. Significance was accepted at p<0.05.

## Results

A total of 561 questionnaires were returned, making the response rate 28%. A total of six questionnaires were excluded due to too much missing information. Thus, 555 questionnaires were included in the analyses. The descriptive characteristics of the study participants are shown in Table 1. More participants were female (55%) and the most common age group was 56–66 years (31%) followed by 46–55 years (27%).

**Table 1:** Characteristics of the 555 study participants in the SWEDIET-2017 study.

	N	%
**Gender**		
Female	306	55
Male	248	45
Other	1	0
**Age (years)**		
20–24	32	6
25–30	39	7
31–36	59	11
37–45	100	18
46–55	152	27
56–65	171	31
**Income (SEK)**		
<10 000	45	8
10–15 000	42	8
15–20 000	37	7
20–25 000	55	10
25–30 000	95	17
30–40 000	128	29
>40 000	120	22
**Education**		
Primary	32	6
Secondary 2 years	69	12
Secondary 3 years	136	25
University <3 years	90	16
University ≥3 years	204	37
Other higher*	17	3
**Employment**		
Full time	372	67
Part time	86	16
Unemployed	16	3
Parental leave	10	2
Student	21	4
Other†	49	9
**Physical activity**‡		
<1/month	103	19
1-3/month	80	15
1/week	94	17
>1/week (ref)	272	50

*Folk high school, advanced vocational education

†Pensioner, daily activity, self-employed, sick leave, in rehabilitation

‡At least 10 min until perspiring

Mean (SD) BMI was 25.4 (4.3) kg/m^2^ and 46% (N = 257) were overweight by self-reported height and weight. However, only 27% (N = 147) considered themselves overweight. In total, 42% of overweight (N = 74) and 90% (N = 70) of obese individuals correctly identified themselves as overweight. Among normal weight individuals, 1% (N = 2) identified themselves as overweight. A higher percentage of men than women were overweight or obese by self-reported data (52% vs. 43%, p = 0.027). However, a lower proportion of men reported being overweight than women (25% vs. 29%, p = 0.297).

**Fig 1:**
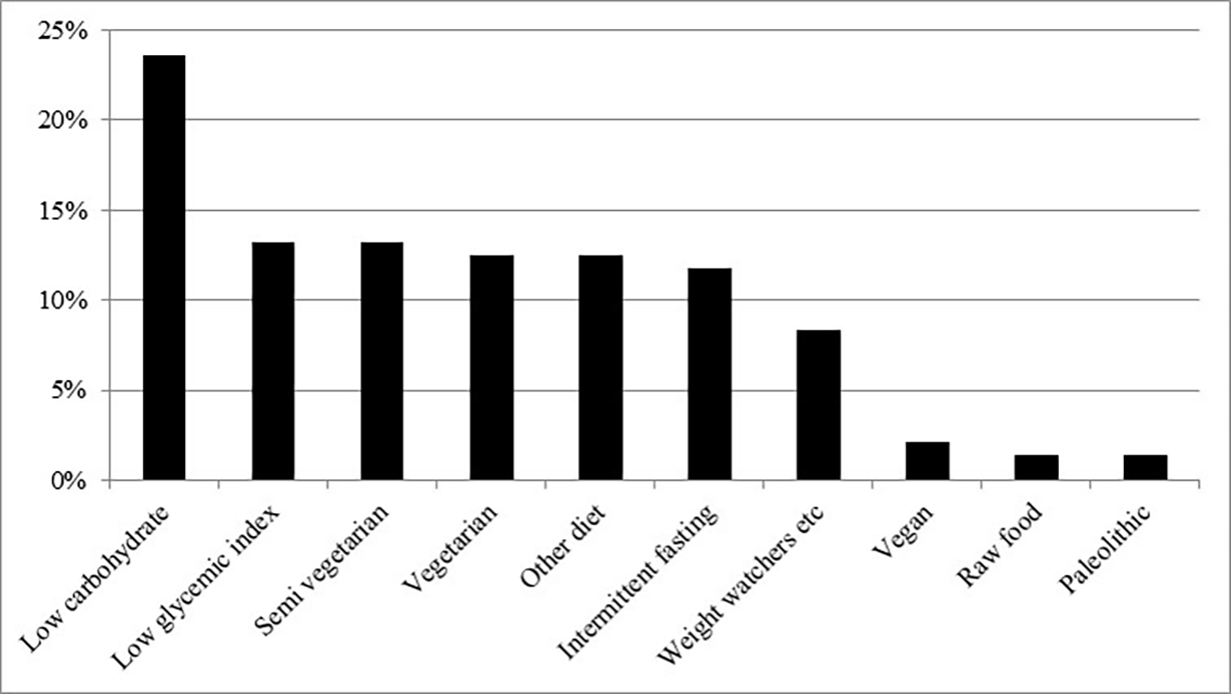
Prevalence of the most common dietary regimens reported in the SWEDIET-2017 survey among the 22% (N = 122) participants who claimed to follow one. SLV; Swedish National Food Agency, Other diet; Most commonly diet after gastric bypass, diabetes diet, the 8 week blood sugar diet and the Mediterranean diet.

**Fig 2:**
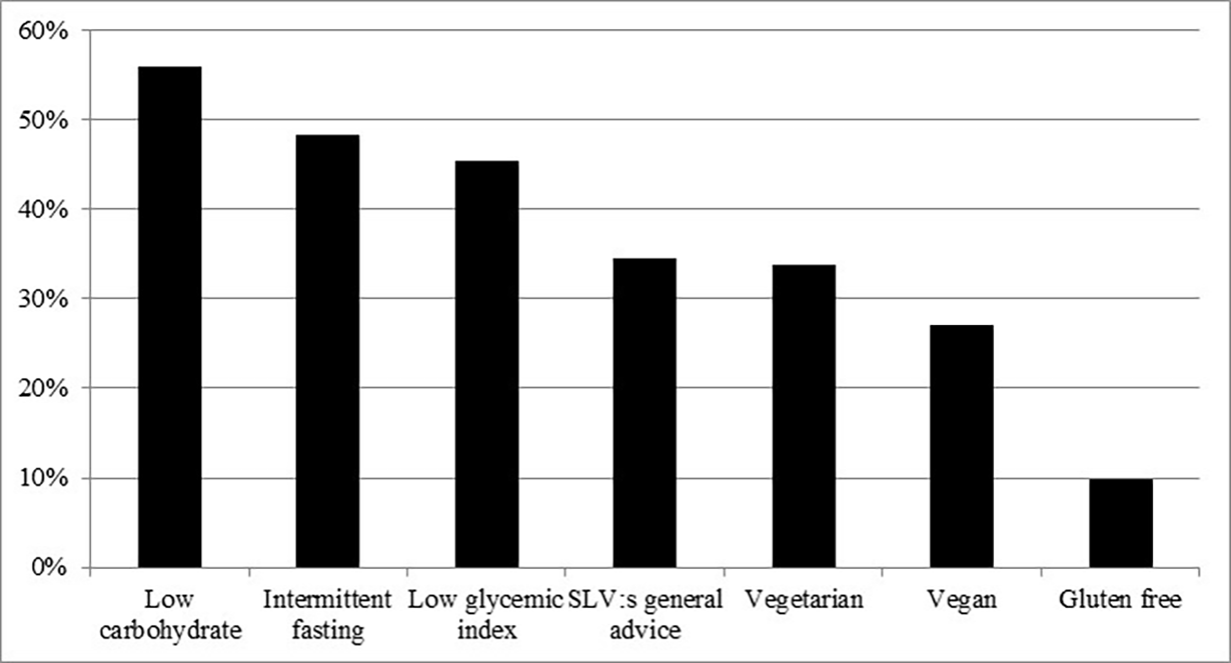
The proportion of the SWEDIET-2017 participants who believes diets are very or partly efficient for weight loss. SLV; Swedish National Food Agency.

**Fig 3:**
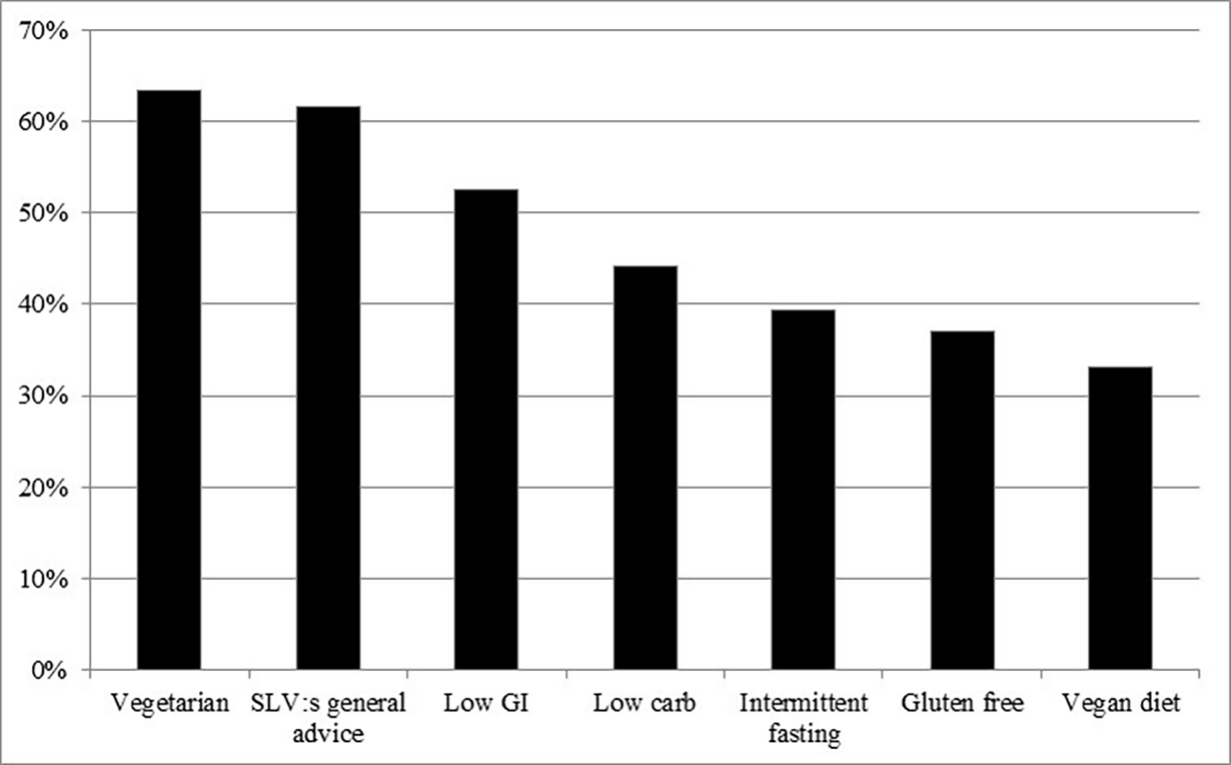
The proportion of the SWEDIET-2017 participants who believes different dietary regimens are very or partly beneficial for health (N = 555). SLV; Swedish National Food Agency, GI; glycemic index.

Of the participants, 41% (N = 228) reported actively trying to lose weight and 3% (N = 16) trying to gain weight. Still, most participants (56%, N = 308) did not actively try to change their weight. In total, 22% of the participants (N = 122) claimed to follow a specific dietary regimen. The most common dietary regimen was low carbohydrate diet (5.9%), followed by low glycemic index diet (3.3%) and intermittent fasting (3.0%) (Fig 1). A total of 40 individuals (7.2%) followed a vegetarian (3.2), semi-vegetarian (3.4%) or vegan diet (0.5%). The dietary regimen that most participants considered as partly or very effective for weight loss was low carbohydrate diet (56%), followed by intermittent fasting (48%), low glycemic index (45%) and the general advice from the Swedish National Food Agency (28%) (Fig 2). The dietary regimen that most participants considered as partly or very beneficial for health was vegetarian diet (63%) followed by the general advice from the Swedish National Food Agency (62%), low glycemic index (53%) and low carbohydrate diet (44%) (Fig 3).

Characteristics significantly related to a desire to lose weight were female gender, higher income, employment status, higher physical activity and higher BMI (Table 2). Age and education level were not related to trying to lose weight.

**Table 2:** Determinants of actively trying to lose weight in multivariable logistic regression analysis (N = 555).

	OR	P	95% C.I. for OR	
			Lower	Upper
**Gender**				
Male (ref)	1.000			
Female	2.151	0.002	1.337	3.460
**Age (years)**		0.719		
56–66 (ref)	1.000			
20–24	0.772	0.656	0.247	2.410
25–30	0.974	0.960	0.340	2.788
31–36	0.629	0.295	0.264	1.498
37–45	1.174	0.647	0.591	2.332
46–55	1.217	0.511	0.677	2.187
**Education**		0.285		
University ≥3 yrs (ref)	1.000			
Primary level	0.657	0.440	0.226	1.908
Secondary level 2 yrs	1.511	0.308	0.683	3.341
Secondary level 3 yrs	1.611	0.137	0.859	3.021
Other higher*	0.995	0.994	0.268	3.685
University <3 yrs	1.722	0.098	0.904	3.279
**Income (SEK)**		0.031		
≥40 000 (ref)	1.000			
<10 000	1.089	0.902	0.281	4.219
10–15 000	0.305	0.069	0.085	1.098
15–20 000	0.427	0.149	0.134	1.357
20–25 000	0.707	0.476	0.273	1.834
25–30 000	0.336	0.006	0.155	0.729
30–40 000	0.895	0.727	0.481	1.666
**Employment**		0.023		
Full-time employment (ref)	1.000			
Part-time employment	0.422	0.024	0.200	0.891
Unemployed	1.273	0.762	0.267	6.084
Parental leave	0.074	0.039	0.006	0.881
Student	0.802	0.789	0.159	4.040
Other†	1.685	0.325	0.596	4.765
**Physical activity**‡		0.007		
>1/week (ref)	1.000			
<1/month	0.317	0.001	0.166	0.607
1-3/month	0.768	0.413	0.408	1.446
1/week	0.843	0.592	0.450	1.577
**BMI (kg/m**^**2**^**)**		<0.001		
BMI <25 (ref)	1.000			
BMI 25–29.9	11.886	<0.001	7.083	19.947
BMI ≥30	19.454	<0.001	9.644	39.240

OR; odds ratio, P; probability, CI; confidence interval, ref; reference

*Folk high school, advanced vocational education

†Most commonly retired

‡At least 10 min until perspiring

Characteristics significantly related to following a specific diet were female gender, higher education and being overweight (Table 3). Age, income, employment status and physical activity level were not related to following a dietary regimen. Among those who claimed to actively trying to lose weight, 32% were following a dietary regimen. Among those reporting following a specific diet, 60% tried to lose weight.

**Table 3:** Determinants of following a specific dietary regimen in multivariable logistic regression analysis (N = 555).

	OR	P	95% C.I. for OR	
			Lower	Upper
**Gender**				
Male (ref)				
Female	2.113	0.003	1.294	3.451
**Age (years)**		0.252		
56–65 (ref)				
20–24	1.237	0.702	0.416	3.677
25–30	1.715	0.249	0.685	4.296
31–36	0.636	0.335	0.254	1.595
37–45	1.473	0.264	0.747	2.904
46–55	0.813	0.532	0.425	1.557
**Education**		0.011		
University ≥3 yrs (ref)				
Primary level	0.771	0.631	0.267	2.228
Secondary level 2 yrs	0.131	0.002	0.036	0.479
Secondary level 3 yrs	0.613	0.135	0.322	1.164
Other higher*	1.503	0.487	0.476	4.740
University <3 yrs	1.309	0.380	0.717	2.390
**Income (SEK)**		0.610		
≥40 000 (ref)				
<10 000	2.313	0.176	0.686	7.805
10–15 000	1.585	0.437	0.496	5.068
15–20 000	1.419	0.545	0.456	4.416
20–25 000	1.567	0.340	0.622	3.946
25–30 000	0.685	0.378	0.295	1.589
30–40 000	1.094	0.785	0.572	2.096
**Employment**		0.657		
Full-time employment (ref)				
Part-time employment	1.066	0.859	0.529	2.146
Unemployed	1.868	0.400	0.436	7.997
Parental leave	1.184	0.831	0.251	5.585
Student	0.445	0.273	0.105	1.891
Other†	1.322	0.595	0.473	3.694
**Physical activity**‡		0.484		
>1/week (ref)				
<1/month	0.925	0.813	0.486	1.762
1-3/month	1.572	0.162	0.834	2.964
1/week	1.015	0.964	0.525	1.965
**BMI (kg/m**^**2**^**)**		0.044		
BMI <25 (ref)				
BMI 25–29.9	1.803	0.021	1.092	2.977
BMI ≥30	0.905	0.791	0.432	1.894

OR; odds ratio, P; probability, CI; confidence interval, ref; reference

*Folk high school, advanced vocational education

†Most commonly retired

‡At least 10 min until perspiring

## Discussion

In this study of 555 Swedish individuals, we found that 46% were overweight or obese based on self-reported weight and height, but only 42% of overweight and 90% of obese individuals correctly identified themselves as overweight. A total of 41% were actively trying to lose weight, and this was associated with female gender, higher physical activity level, overweight or obesity and higher socioeconomic position (higher income and employment). In total, 22% followed a specific diet, and this was associated with female gender, higher education and overweight.

The results of this study show that almost half of the respondents were trying to lose weight. In addition, almost every fourth participant reported following a specific dietary regimen, which is higher than the 9% described in the national Swedish dietary survey from 2010–2011 [16]. The results of the current study further suggest that women diet and try to lose weight to a larger extent than men, despite overweight being less common among women than among men. This is in line with findings from the national Swedish dietary survey that revealed lower self-reported BMI and less overweight among women than among men [16]. Previous studies have demonstrated that it is more common for women to try to lose weight [10, 11], which concurs with our results. It is not known however, if women are less overweight because of their weight management, or if they try to lose weight despite a lower BMI.

Higher socioeconomic position was related to both dieting (education) and a desire to lose weight (income and employment status). This is supported by previous findings that lower socioeconomic position is associated with obesity [4]. Higher levels of physical activity were related to trying to lose weight, which might reflect an attempt to facilitate weight loss. The finding that higher BMI is associated with a desire to lose weight has been indicated [19] and is reasonable, since much emphasis is placed on the importance of a normal BMI for health [20].

The most common dietary regimen reported was low carbohydrate diet, followed by low glycemic index diet and intermittent fasting. Low carbohydrate diets such as the low carb-high fat diet gained much popularity in Sweden in the early 2000’s [17] and our results suggest that it is still common, and viewed by most participants as effective for weight loss. However, a smaller proportion regarded low carbohydrate diet as beneficial for health (44%) than as effective for weight loss (56%). The dietary regimens regarded as beneficial for health by the highest and lowest proportion of participants were vegetarian diet (63%) and vegan diet (33%), respectively.

### External validity

The prevalence of overweight in this study is comparable to the general Swedish population where approximately 50% are overweight or obese by self-reported data [1]. Participants between the ages of 20–65 years were recruited to participate in the study. This decision was made in order to recruit those who were likely to live in an independent household, with autonomy over their food choices. The results are therefore not transferable to individuals under the age of 20 or over the age of 65. Mean monthly income in 2016 in Sweden was 32 800 SEK and 25% earned less than 25 000 SEK per month [21]. Most participants in the current study had an income of 30–40 000 SEK per month and 33% earned less than 25 000 SEK. It therefore seems that the study sample have a slightly lower income than the average Swedish population. However, the proportion of the study sample (37%) with at least 3 years higher education was higher than in the average Swedish population (27%) [22]. The 555 people who participated in this study are therefore slightly more educated but seem representable of the general Swedish population in terms of income and prevalence of overweight. Few data exist on how common specific dietary regiments are in Sweden, but the proportion in our sample who follow general vegetarian or vegan diets is in line with previous national data [16].

### Strengths and limitations

The strengths of this study are the randomly selected population-based study sample, the relatively high response rate for a questionnaire-based study design using postal delivery without reminders (28%), high completion rate among responders (99%) and that the questions enabled detailed statistical analysis of the research questions. The limitations are associated with the nature of a questionnaire-based cross sectional study design; only possible to study associations, self-report data and the inability to make follow-up questions to probe further. The questionnaire used in this study has not been validated. It is possible that some participants believe to adhere to e.g. low carbohydrate or glycemic index diet, but have limited knowledge of what that entails. In addition, physical activity was only defined as exercise, but this was deemed sufficient in the current study of behaviors relating to overweight and dieting and not to dietary intake or energy expenditure.

## Conclusions

In conclusion, 41% of Swedish participants actively tried to lose weight and 22% reported following a specific dietary regimen. Female gender, higher BMI and higher socioeconomic position were associated with attempts at weight loss and following a specific diet. This could indicate that the socioeconomic disparities in health are further exacerbated, as overweight individuals with poor socioeconomic position might be more likely to remain overweight.
